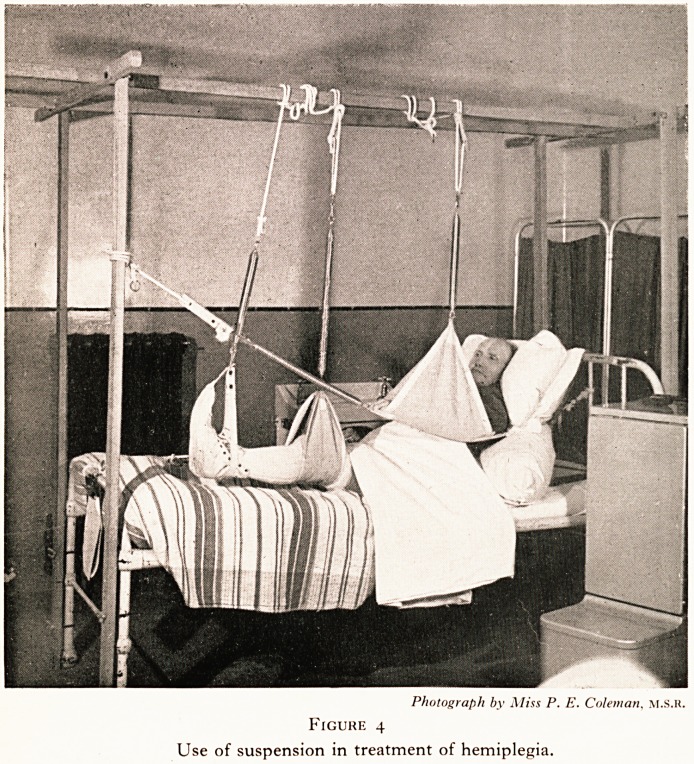# Modern Developments

**Published:** 1951-04

**Authors:** T. S. Wilson


### GERIATRICS.
### II : MODERN DEVELOPMENTS

BY

T. S. WILSON, M.D.

---

The problem of infirmity in old age can no longer be disregarded. The problem is, of course, that of the elderly sick who formerly remained at home as invalids, or gravitated to the chronic wards of hospitals or Public Assistance institutions. Geriatrics is primarily concerned to-day with the treatment of disease and the restoration of as great a degree of personal independence as possible to these elderly patients. Many of the disorders of this age-group were regarded with fatalism in the past, often unjustifiably: and so received less than their fair share of consideration either with regard to their cause or prevention. Frequently the age of the patient in itself was considered an adequate explanation of a variety of ills which were lightly dismissed on that account.

### Incontinence

For example, the elderly incontinent patient in the acute ward received short shrift and was usually rapidly moved to the nether regions of the hospital. Reference to the scanty literature dealing with incontinence does not indicate any considerable advance in thought during the course of the past few centuries and in comparatively recent years we find the view expressed that it is " probably the result of senile weakness of the musculature concerned and not in a disorder of the nervous controlling mechanism ". Yet we now know that incontinence of urine in old people is not necessarily an irreversible condition and that, by re-education and attention to the underlying pathology, it can frequently be relieved. Cystometric methods have shown us that far from being the result of senile muscular weakness, the vast majority of these cases are caused by an *over-activity* of the neuromuscular bladder mechanism, which results in uncontrolled bladder contractions.

This bladder spasm results from the interplay of two factors: (i) Conditions causing local irritability of the bladder reflex, the commonest being a chronic urinary infection. (ii) Conditions which impair the (cortical) inhibitory control of the bladder reflex—cerebral vascular lesions, cerebral degeneration, constitutional disturbances such as severe anaemia and the like. Both these factors are open to therapeutic attack, the first by relieving local irritant condition, the second by re-education in bladder control. (Figs. 1 and 2.) I merely mention this to illustrate the point that it is essential to consider these disabilities in more detail, to investigate them fully and to try and get at the fundamental causes. The time is past when we might regard them as irrevocable penalties of advancing years.

### Inactivity

Perhaps one of the most important advances in the treatment of the elderly sick is the recognition of the evil effects of prolonged inactivity. The chain of infirmities which may result from such inactivity alone includes muscular weakness and atrophy, fixation of joints, postural contractures and the mental apathy and indifference which is so characteristic. Because of the importance of ensuring maximum activity, physical rehabilitation plays a big part in modern geriatric treatment. Suspension therapy has a particular application to this age-group. In the elderly arthritic patient, in addition to the actual condition of

FIGURE 1

Cystometry: periodic bladder spasm

FIGURE 2
Gystometry: restored bladder control

PLATE III

*Photograph by Miss P. E. Coleman,* M.S.R.

FIGURE 3

Suspension methods for the elderly arthritic.

PLATE IV

*Photograph by Miss P. E. Coleman,* M.S.R.

FIGURE 4

Use of suspension in treatment of hemiplegia.

the joint and periarticular tissues, mobility is hampered by the weight of the limb, by muscular enfeeblement, by friction and by subconscious muscle guarding, which occurs if pain is likely on movement.

In suspension the effects of gravity are counteracted, friction is eliminated and as movement is under the control of the patient, muscular guarding is minimized. (Plate III, Fig. 3). The result is that one is able to get a greatly increased range of movement and the patient is spurred on to greater efforts, with improvement in muscle power and joint function. In due course, the patient progresses to exercises against spring resistance, the springs being of varying strengths which can be graded according to the individual patient or muscle group concerned.

### Hemiplegia

It is in the treatment of the hemiplegic patient that suspension therapy has the greatest application. The paralysed limbs are suspended by slings and springs from a Balkan beam. (Plate IV, Fig. 4).

This has the advantage that the limb is under observation, and one can be sure it is not assuming undesirable postures under the bedclothes. There appears to be a decreased tendency to the development of spasticity in such a limb suspended from the outset. The patient can be encouraged to start active movements at a very early stage and the feeblest of muscular effort will produce a visible result.

Such a patient is allowed to sit up in a chair as soon as possible, usually a matter of several weeks or less, and should start standing exercises at the foot of the bed. These consist merely of the patient sitting in a chair at the foot of the bed, holding on to the bedrail, raising himself to his feet and lowering himself into the chair once again. They are simple exercises which, if carried out assiduously, have excellent results in strengthening musculature and improving stability. From this the patient proceeds to actual walking exercises, either in a walking-chair or a walking-alley. For the arm, pulley exercises are carried out, but recovery in the arm is usually much slower and more incomplete than in the leg.

Untreated hemiplegic patients are a considerable burden on the hospital service. McEwan, in his survey of Bradford chronic hospitals, found that about 15 per cent. of patients were there as a result of a hemiplegia. Many of them had been in hospital a considerable time and the average duration of stay was over two years. By active treatment, especially in the early stages, this burden can be drastically reduced. Of 370 consecutive admissions to Barncoose Hospital, 21 per cent. were hemiplegics. Eighty per cent. of these hemiplegics were classified as severe lesions and the cerebral lesion was often associated with other gross changes which in many cases was the cause of death or prevented intensive remedial measures. Of these patients, one-third died; one-fifth were irremediable, and remained as chair or bedfast invalids; but nearly half became ambulant again. (The average time in hospital was three and a half months.) From these figures it is apparent that of those who survive, over two-thirds can become ambulant again.

## Figures and Tables

**Figure 1 f1:**
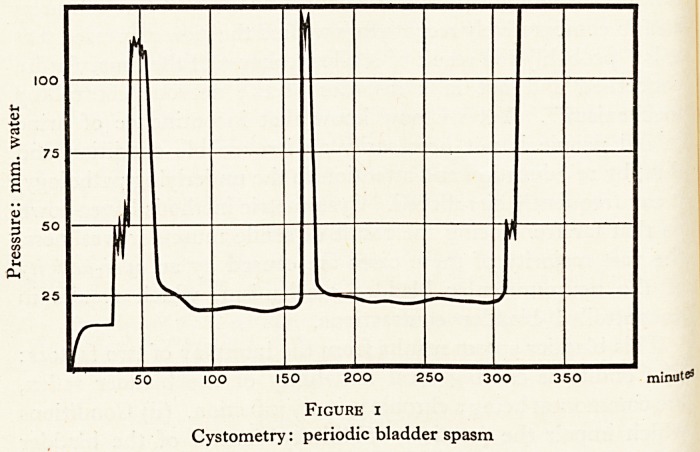


**Figure 2 f2:**
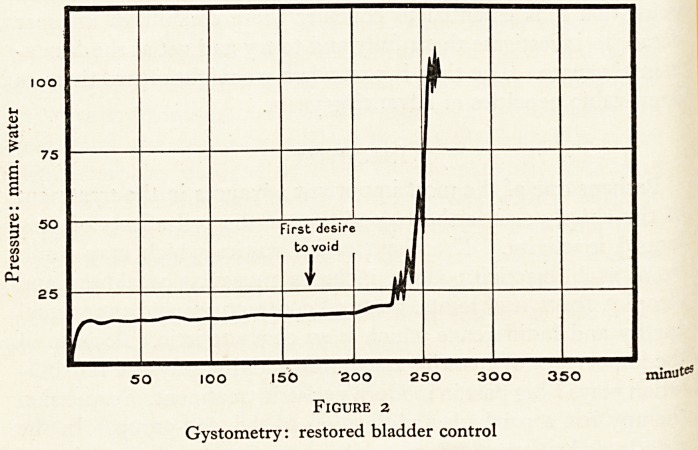


**Figure 3 f3:**
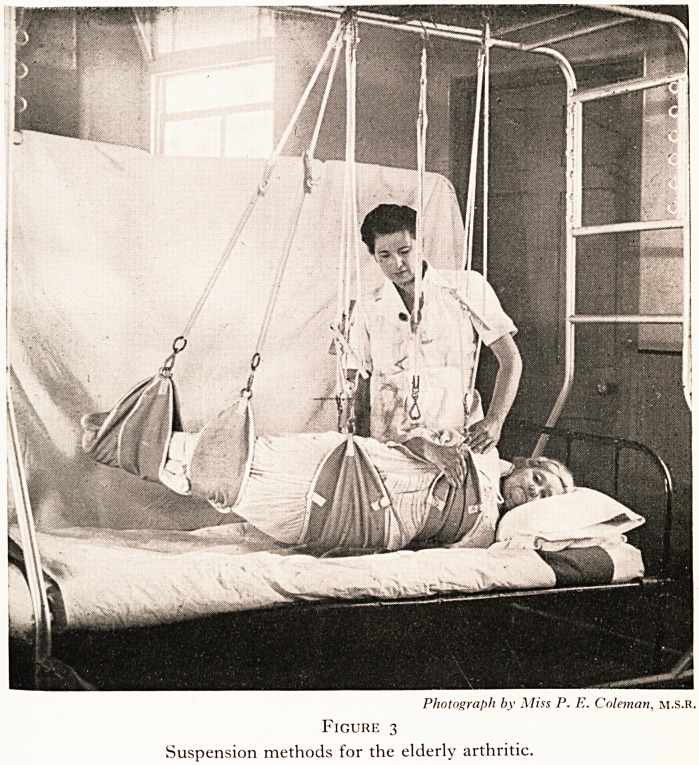


**Figure 4 f4:**